# A Traumatic Direct Inguinal Hernia from Pelvic Ring Disruption

**DOI:** 10.1155/2018/5392430

**Published:** 2018-07-10

**Authors:** Kevin L. Chow, Eduardo Smith-Singares, James Doherty

**Affiliations:** ^1^Department of Surgery, University of Illinois at Chicago, MC 958, 840 South Wood Street, Suite 376-CSN, Chicago, IL 60612, USA; ^2^Department of Surgery, Division of Trauma Critical Care, Advocate Christ Medical Center, 4440 W. 95th Street, Suite 183, South Oak Lawn, Chicago, IL 60453, USA

## Abstract

**Introduction:**

Pelvic fractures usually involve a high-energy traumatic mechanism and account for approximately 3% of all blunt traumatic skeletal injuries. Additional musculoskeletal injuries are found in over 80% of unstable pelvic fractures. Traumatic abdominal wall hernias (TAWHs) are a rare entity, and traumatic inguinal hernias (TIHs) associated with open-book pelvic fractures have not been described previously.

**Case Presentation:**

We present the case of a 45-year-old male motorcyclist involved in a collision resulting in a traumatic direct inguinal hernia due to abdominal wall disruption from an open-book pelvic fracture. He underwent a combined operation with an open reduction and internal fixation (ORIF) of his pelvic fracture and an abdominal wall reconstruction with a modified Stoppa technique utilizing mesh for his hernia.

**Discussion:**

This is a unique presentation of a TIH due to an open-book pelvic fracture after blunt abdominal trauma. The formation of TAWH is typically from a combination of local tangential shearing forces and a sudden rise in intraabdominal pressures damaging the muscle, fascia, and peritoneum while the skin remains intact. In patients without hollow viscous injuries and gross contamination, these hernias can be repaired safely with mesh in the acute setting simultaneously with pelvic reduction.

## 1. Introduction

Acute traumatic abdominal wall hernias (TAWHs) are rare but can be potential injuries seen after blunt trauma. First reported in 1906, TAWHs are defined by three criteria: (1) herniation through disrupted musculature and fascia after sufficient trauma, (2) no evidence of skin penetration, and (3) no evidence of a prior hernia defect [1, 2]. Traumatic inguinal hernias (TIHs) fall under that definition, but to our knowledge, a case resulting from an open-book pelvic fracture has not been described.

Pelvic fractures usually involve a high-energy traumatic mechanism and account for approximately 3% of all blunt traumatic skeletal injuries. Additional intraabdominal and musculoskeletal injuries are expected to be found in over 80% of unstable pelvic fractures given the associated high-energy trauma [3]. TAWH is a rarely reported consequence with fewer than 40 cases of bowel herniation associated with traumatic pelvic fractures reported over the past century [4, 5].

In this article, we summarize the presentation of an acute TIH due to an open-book pelvic fracture after blunt abdominal trauma. This injury was managed with a simultaneous open reduction and internal fixation (ORIF) of the pelvis and abdominal wall reconstruction with mesh. This case report was written following the recommendations for the conduct, reporting, editing, and publication of scholarly work in medical journals (contained in the ICJME statement, http://www.icmje.org/), the work of the Equator Network (http://www.equator-network.org/) and the SCARE checklist [6].

## 2. Case Presentation

**Figure 1:**
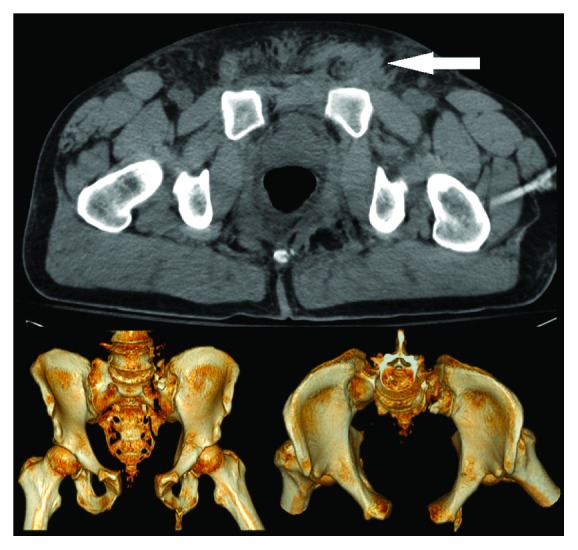
CT Reconstruction demonstrating open-book pelvic fracture. The white arrow demonstrates the inguinal hernia.

A 45-year-old male motorcyclist with a history of hypertension, hyperlipidemia, and coronary artery disease was brought to the emergency department after being struck by another car on the highway at speeds of at least 40 miles per hour. Upon presentation, the patient was evaluated using Advanced Trauma Life Support (ATLS) principles. He had a patent airway on arrival and was breathing spontaneously on room air. His initial heart rate was 87 beats per minute, and his blood pressure was 124/63 mmHg without signs of significant hemorrhage. He had an initial Glasgow coma score (GCS) of 15 with equal and reactive pupils. The patient admitted to consuming alcohol and had a serum alcohol of 243 mg/dL. A later CT of the head demonstrated a subcutaneous hematoma without any intracranial abnormalities. His remaining physical examination revealed left lower quadrant abdominal pain without signs of peritonitis, ankle deformities bilaterally, pain with hip range of motion, and blood at the urethral meatus. Given his physical examination findings, subsequent imaging confirmed an unstable pelvic fracture with diastasis of the *symphysis pubis* of 6 cm, widening of the left sacroiliac joint, a left ischial pubic ramus fracture, and a urethral injury (Figure 1). He also had a left ankle dislocation and a right compound fracture of the distal tibia and fibula. No intraabdominal injuries were identified on CT imaging of the abdomen. The pelvis was stabilized with a binder by the orthopedic surgeons with subsequent emergency irrigation, debridement, and open reduction and internal fixation (ORIF) of the open ankle fracture as well as reduction of the left ankle dislocation. He was extubated after the procedure and monitored in the ICU while the remaining preoperative medical workup was completed including X-rays and CT scans with 3D reconstructions of the pelvis reconstructions. A hydromorphone patient-controlled analgesia (PCA) pump was utilized for pain control.

**Figure 2:**
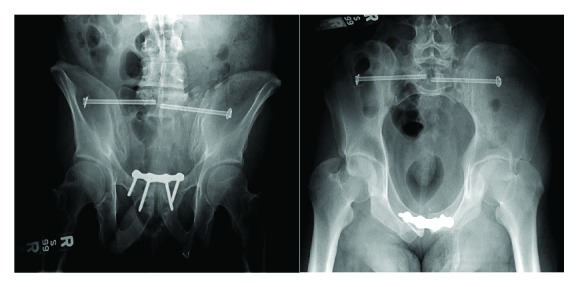
Postreduction X-rays.

On hospital day 2, the patient was deemed fit for surgery and was taken to the operating theater for a combined operation by the orthopedic surgeons for ORIF of the pubic diastasis, sacral fracture, and sacroiliac joint followed by the trauma surgeons to reconstruct the abdominal wall and inguinal canal. The trauma team performed the exposure of the pubic symphyseal region and the pubic diastasis. A Pfannenstiel incision was made, and the planes were dissected exposing the left spermatic cord. The orthopedic team then performed a gentle open reduction of the pubic diastasis taking care to ensure that the bladder and urethra were not incarcerated. The Asnis III cannulated screw system and a Matta pubic symphyseal plate (Stryker GmbH, Switzerland) were utilized under C-arm fluoroscopic guidance with appropriate alignment of the AP and inlet and outlet pelvis views. Once the Mata plate was in place and the orthopedic reduction was completed, we proceeded to reconstruct the anterior abdominal wall. Since the Cooper ligament was destroyed, it was dissected to allow direct visualization of the pubic rami. The abdominal wall defect was measured to be 10 × 12 cm. We then used a modified Stoppa technique by placing the 6 × 6 in Prolene mesh under the damaged internal inguinal ring, making sure the spermatic cord on the left side was not injured or pinched, securing it in place using sutures, including direct suturing to the periosteum of the repaired pubic symphysis and the plate as needed. The medial borders of the mesh were tucked inside the opened rectus sheath on the right side and secured laterally with fires of a 5 mm Covidien Endotack (Medtronic, MN, USA) to the remnants of the conjoint ligament. The midline was then repaired with sutures, including the mesh as reinforcement. The patient did well postoperatively with postreduction films demonstrating appropriate alignment (Figure 2). He was discharged to rehab on postoperative day 5. There were no recurrences during the follow-up period of 10 years.

## 3. Discussion

True acute TIHs are a rare entity that falls within the category of TAWH with some believing them to be a “myth” [7]. TAWHs are a rare sequelae of blunt abdominal trauma with an estimated incidence of 0.07% in a large single-center retrospective analysis of CT scans performed for blunt trauma [8]. The mechanism of TAWH typically involves a combination of local tangential shearing forces and a sudden rise in intraabdominal pressures resulting in damage or disruption to the underlying muscle fibers, fascia, and peritoneum. The skin may or may not be involved as its inherent elasticity is protective [2].

These hernias occur most frequently after motor vehicle collisions (40%) and motorcycle collisions (16%). The clinical presentation of TAWH typically involves a tender soft tissue mass on the abdomen (31%) or an associated ecchymosis (49%). They can present acutely in the case of concomitant intraabdominal injuries and peritonitis or evisceration. A delayed presentation is also possible with muscle spasm from pain following trauma initially masking the defect. Delayed herniation is also a possibility following the weakening of the abdominal wall from a hematoma or wound infection [5]. The CT scan is the diagnostic modality of choice and allows for the detection and grading of abdominal wall injuries, first described by Liasis et al. and Dennis et al. [2, 8].

Early reports of patients with TAWH recommended early operative exploration and repair due to the high risk of concomitant intraabdominal injuries and potential for incarceration [9, 10]. More recent studies have debated that recommendation by demonstrating that in the absence of concurrent intraabdominal injuries, delayed repair or a laparoscopic approach can be considered [10–12]. However, the natural history of undetected hernias is progressive enlargement that can lead to bowel incarceration and strangulation. Therefore, early abdominal wall reconstruction with tension-free repairs remains the standard. The use of mesh repairs is well recognized for its ability to minimize recurrence rates but must be weighed against the risk of infection particularly with hollow viscus injuries and abdominal contamination [13].

We presented the unique presentation of a TIH due to an associated open-book pelvic fracture after blunt abdominal trauma. The hernia was diagnosed based on CT imaging, and there were no other concomitant intraabdominal injuries found. It was then repaired with a tension-free, modified Stoppa mesh technique during the same operation as the pelvic ORIF. The patient had a good outcome without recurrence during the follow-up period of 10 years.

## 4. Conclusions

In summary, the management of TAWH and TIH is complicated given the high association with other concomitant intraabdominal and musculoskeletal injuries particularly with pelvic fractures. Here, we have shown that for selected patients, without severe intraabdominal injuries or gross contamination, it is possible to perform the abdominal wall and inguinal canal reconstruction with a tension-free, modified Stoppa mesh technique and reduce the pelvis simultaneously in the acute setting with durable results.
